# Thank You to the Journal's Peer Reviewers

**DOI:** 10.1176/appi.prcp.20250125

**Published:** 2025-11-10

As Interim Editor‐in‐Chief, I thank our reviewers for undertaking the important and necessary work of conducting peer reviews of submitted manuscripts. Our reviewers provide a valuable service to the field through their efforts, as peer review is the bedrock of quality control. Identifying the strengths and weaknesses of every submission—and, through thoughtful suggestions and comments, guiding authors to ways to improve their manuscripts—enhances the rigor and impact of manuscripts we publish. The labor of peer reviewers too often goes unrecognized, despite the fact that reviewers donate their valuable and scarce time to this work. I appreciate the dedication and professionalism of all of our reviewers. Thank you for helping ensure that the publications in this journal meet the standards the field should expect.

—Laura B. Dunn, M.D.

**REVIEWERS**

Jesús Alberdi Sudupe

Jenifer Allsworth

Yuen‐Siang Ang

Gerard Anmella

Ann Annis

Jacob Appel

Tagbo Arene

Anees Bahji

Catherine Barry

Kate Bentley

Matthew Boone

Chelsey Bull

Christine Chapais

Joseph Chih‐Chien Chen

Erika Clark

Sabrina Correa da Costa

Francine Cournos

Brian Daly

Tisha Deen

Michele Easter

Xiaoduo Fan

Blossom Fernandes

Laura Fochtmann

David Fogelson

Ariadna Forray

Jane Gagliardi

Amir Garakani

Gryan Garcia

Clemente Garcia‐Rizo

Julie Goldstein Grumet

Christine Gould

Kathleen Grubbs

Ozlem Gunal

Elizabeth Hancq

Amy Harrington

Allyson Harrison

Diego Hidalgo‐Mazzei

Claudine Higdon

Jacqueline Huber

Fabio Idrobo

Lee Isaac

Isha Jalnapurkar

Nev Jones

Maria Kamusheva

Jinny Kim

Franklin King IV

Brian Kirkpatrick

Carolina Klein

Junko Koike

Steven L. Lancaster

Daniela Lemmo

Jessie Lenagh‐Glue

Álvaro López‐Díaz

Ivan David Lozada Martinez

Rachele Mariani

Carla Marienfeld

John Markowitz

Marianna Mazza

Meera Menon

Alexander Missner

Steely Smith Mollee

John Monahan

Ana Montes Montero

Fatima Motiwala

Claire Nollett

Anthony O'Brien

Amy O'Shea

Barton Palmer

Pallav Pareek

Shreedhar Paudel

Jennifer Payne

Matthew Peters

Katie Prizeman

H. Paul Putman

Paul Putnam

Aneesh Rahangdale

Veronica Raney

Paula Ravitz

Shona Ray‐Griffith

Eva Regel

Abigail Richison

Kathryn Ridout

Abid Rizvi

Laura Rohm

Kelly Rootes‐Murdy

Ashok Roy

Neeti Rustagi

Susan Schultz

Yelizaveta Sher

Greg Simon

Gregory Simon

Brian Skehan

Mark Solms

Eli Somer

Kirsty Sprange

Drozdstoy Stoyanov

Sandarsh Surya

Leonard Swanson

Sarah Taha

S. Darius Tandon

Aslı Tok Özen

John Torous

Francesco Tramonti

Baeleigh VanderZwaag

Mytilee Vemuri

Heidi Waters

Colin Whitworth

Hannah Williams

Xihan Yang

Hsueh‐Han Yeh

Caner Yesiloglu

Taylor Young

Shani Zilberman‐Itskovich

